# Interleukin-35 promotes progression of prostate cancer and inhibits anti-tumour immunity

**DOI:** 10.1186/s12935-020-01583-3

**Published:** 2020-10-07

**Authors:** Jialin Zhu, Yan Wang, Dai Li, Haonan Zhang, Zhi Guo, Xueling Yang

**Affiliations:** 1 Department of Ultrasound Diagnosis and Treatment, Tianjin’s Clinical Research Center for Cancer, Key Laboratory of Cancer Prevention and Therapy, National Clinical Research Center of Cancer, Tianjin Medical University Cancer Institute and Hospital, Tianjin, 300060 China; 2 Department of Radiology, The Second Affiliated Hospital of Zhejiang University School of Medicine, Hangzhou, 310000 China; 3 Department of Geriatrics, Laboratory of Neuro-Trauma and Neurodegenerative Disorders, Tianjin Geriatrics Institute, Tianjin Medical University General Hospital, Tianjin, 300000 China; 4 Department of Interventional Therapy, Tianjin’s Clinical Research Center for Cancer, Key Laboratory of Cancer Prevention and Therapy, National Clinical Research Center for Cancer, Tianjin Medical University Cancer Institute and Hospital, Huan Hu West Road, Tianjin, 300060 China

**Keywords:** Interleukin-35, Prostate cancer, Proliferation, Angiogenesis, Anti-tumour immunity

## Abstract

**Background:**

Interleukin-35 (IL-35) has been reported to play an important role in the progression of cancers. The role of IL-35 in prostate cancer (PCA) is not well understood. In this study, we investigated the effects of IL-35 on PCA and its immunoregulatory effect on PCA.

**Methods:**

The protein and mRNA expression of IL-35 in PCA cells was detected by western blot and RT-PCR. The invasion and migration of cells were detected using transwell and wound‐healing assays. A CCK-8 assay was conducted to observe cell proliferation. In vivo, IL-35 plasma concentration was test by enzyme-linked immunosorbent assay. The role of IL-35 in tumour cell proliferation and angiogenesis of mice was detected by immunohistochemical stains. The mouse survival and tumour volumes were calculated, and lung metastasis rate was detected by HE staining. The modulatory effects of IL-35 on myeloid-derived inhibitory cells (MDSCs), regulatory T cells (Tregs), CD4+ T cells and CD8+ T cells from PCA mice were investigated by immunohistochemical stains and flow cytometry.

**Results:**

High levels of IL-35 significantly promoted the migration, invasion and cell proliferation of PCA cells in vitro. IL-35 was associated with tumour growth, metastasis and poor prognosis in PCA mice. Additionally, high levels of IL-35 significantly increased the proportions of MDSCs and Tregs and decreased the proportions of CD4+ and CD8+ T cells in the spleen, blood and tumour microenvironment. The IL-35 neutralizing antibody played the opposite role.

**Conclusions:**

IL-35 contributed to the progression of PCA through promoting cell proliferation and tumour angiogenesis. IL-35 might limit the anti-tumour immune response by upregulating the proportions of Tregs and MDSCs and by reducing the proportions of CD4+ and CD8+ T cells. IL-35 might serve as a novel therapeutic target for PCA.

## Background

Prostate cancer (PCA) is the second most common malignant tumour and the fifth leading cause of cancer-associated mortality in men worldwide [1]. Patients with localized PCA are usually treated with surgery or radiotherapy. However, 20–40% of patients receiving radical prostatectomy and 30–50% of patients receiving radiation therapy will have a recurrence that develops into metastatic disease [2]. At present, there is no ideal treatment for metastatic castrated prostate cancer, and most of the patients have poor prognoses [3]. Therefore, it is urgent to develop a more effective treatment.

In recent years, bio-immunotherapy has become a new therapy that has attracted increasing attention. Studies have shown that some tumour cells evade immune recognition and killing by activating the negative stimulus signal and by regulating the inhibitory immune response [4]. In addition, tumour cells can recruit immunosuppressive cells, such as regulatory T cells (Tregs) and myeloid-derived inhibitory cells (MDSCs), and mediate the release of immunosuppressive factors directly or indirectly. Immunosuppressive cells jointly promote the occurrence and development of the immunosuppressive tumour microenvironment and significantly promote tumour progression. Therefore, some cytokines with immunosuppressive functions, such as transforming growth factor (TGF)-β, interleukin (IL)-6, and IL-35, have attracted great attention [5, 6].

IL-35 is a newly discovered immunosuppressive cytokine that consists of EBI3 and P35 subunits and belongs to the IL-12 cytokine family [7]. This family has become the focus of tumour biological immune related domain research [8]. Some research has shown that IL-35 is highly expressed in a wide range of tumour tissues, which could promote tumour angiogenesis and inhibit anti-tumour immunity [9, 10]. In many different types of malignant tumours, plasma IL-35 levels are closely related to tumour stage, tumour size and lymph node metastasis [11, 12]. High expression of IL-35 in tumour tissues and increased expression of IL-35 in plasma suggest poor prognosis of patients [13–16].

The results of current clinical studies on PCA showed that the average concentration of serum IL-35 in patients with PCA was significantly higher than that in healthy controls, indicating that IL-35 may be involved in the tumourigenesis of PCA [17, 18]. Our previous research shows that increased expression of IL-35 in the plasma and tumour tissues may contribute to the progression and metastasis of PCA patients [17]. However, the expression of IL-35 in different PCA cell lines and the mechanism of how IL-35 effects progression and metastasis of PCA have not been reported. The purpose of this study was to detect the expression of IL-35 in different prostate cancer cell lines, to detect the effects of IL-35 on the biological behaviour of PCA cells, and to explore the effects of IL-35 on tumour proliferation, angiogenesis, metastasis and immune cell populations in PCA mice.

## Materials and methods

### Cell culture

The human PCA cell lines PC-3, DU145, LNCaP and mouse PCA cell line RM-1 were purchased from Shanghai Institutes for Biological Science of the Chinese Academy of Sciences (Shanghai, China). These cells were cultured in Roswell Park Memorial Institute-1640 medium (RPMI-1640) (Gibco, Carlsbad, CA, USA) or F-12K nutrient medium with 10% foetal bovine serum at 37° C in a humidified atmosphere of 95% air and 5% CO_2_. To investigate the regulatory effect of IL-35 on RM-1 cells, the cells were divided into NC (double distilled water as a negative control), IL-35 (recombinant IL-35 protein, 200 ng/ml, diluted by double distilled water), Scramble (PBS, as a control group for the IL-35 NA group), and IL-35 NA (anti-IL-12A antibody, 200 ng/ml, diluted by PBS) groups.

### Western blot analysis

For protein extraction, cells or tumour tissues were lysed in RIPA buffer supplemented with a protease inhibitor cocktail (Sigma, USA). A total of 30 mg protein lysate was separated by SDS–PAGE, and then the target proteins were detected by western blot analysis with antibodies against EBI3 (ab83896, Abcam) and p35 (ab66064, Abcam). β-Actin (ab8227, Abcam) was used as an internal control.

### RNA extraction and real-time polymerase chain reaction (RT-PCR)

Total RNA of the cells was extracted with TRIzol reagent (Invitrogen, USA) according to the manufacturer’s instructions. Then, the quantity and purity of RNA was determined by absorbance on a microplate reader (Sunnyvale, USA) at 260 nm and 280 nm. Samples with ratios from 1.7 to 2.0 were accepted for the next reverse transcription reaction. The mRNA was reverse transcribed to single-stranded complementary DNA by using a RT-PCR system (TaKaRa, Japan). Real-time fluorescent quantitative PCR was used to analyse the cDNA levels. The primer sequences are shown in Table1. IL-12 is composed of p35 and p40, and IL-27 is composed of p28 and EBI3. The detection of p40 and p28 was to exclude the effects of IL-12 and IL-27.

**Table 1:** Primer sequences used for RT-PCR

Genes	Forward sequence	Reverse sequence
EBI3	GATCCGTTACAAGCGTCAGG	CTCAGTTCCCCGTAGTCT
p35	CTCCCTTGAAGAACCGGAT	ATCAATAGTCACTGCCCGAA
p40	CCCTGACATTCTGCGTTCA	AGGTCTTGTCCGTGAAGACTCTA
p28	CAGACGGCAGGCGACCTT	TGACTGTGAACTCCCTCCGC

### Cell invasion assay

The invasive ability of cells was detected in 24-well transwell chambers with 8.0 mm pore inserts pre-coated with Matrigel. For this assay, the tumour cells were digested and resuspended in FBS-free RPMI-1640 culture medium. A total of 100 µl (1.5 × 10^5^ cells per ml) of tumour cells was seeded into the upper chamber. The basolateral chambers were supplemented with 60 µl complete culture medium (RPMI-1640 with 20% FBS), and the cells were incubated at 37 °C in 5% CO_2_ for 24 h. For each insert, the invading cells in five random fields of 200× magnification were counted. Each experiment was performed in triplicate.

### Cell migration assay

The migration ability of prostate cancer cells was detected using a wound healing assay. First, RM-1 cells were seeded in 6-well plates at approximately 80% confluence. After that, a wound was made in the centre of each well using a 10 μL pipette tip. Each well was washed with PBS twice and added to media containing 1% FBS and different solutions (double distilled water, rhL-35 protein, PBS, or anti-IL-12A antibody). At 0 h and 48 h, images of the wound area of each well were captured. Cell migration was calculated as the area of cell migration compared to the area of the initial wound. All experiments were repeated three times.

### Cell proliferation assay

Cell proliferation was evaluated by cell-counting kit-8 (CCK-8) (Dojindo, China) according to the manufacturer’s manual. In brief, 100 μL of cell suspension (5000 cells) was seeded into each well of a 96-well plate. The plate was incubated for the indicated time periods (12, 24, 36, 48, 60 and 72 h) in an incubator at 37 °C in 5% CO_2_, and 10 μL of CCK-8 solution was added to each well of the plate. The plate was again incubated for 1 h, and the absorbance at 450 nm was measured using a microplate reader. Each time point was repeated in three wells, and the experiment was independently performed three times.

### Animal study

This study was evaluated and approved by the Ethics Committee of the Tianjin Medical University Cancer Institute and Hospital, and conducted by skilled experimenters. All applicable international, national, and institutional guidelines for the care and use of animals were followed. Male 5-week-old C57BL/6 mice (SPF Biotechnology Co., Ltd., Beijing, China) were housed in specific pathogen-free conditions. For evaluation of tumour growth in vivo, 1 × 10^6^ cells were suspended in 100 μl PBS and injected subcutaneously into the inguinal region of C57 mice. Tumour growth was monitored every 3 days, and a growth curve was drawn from these data. Tumours were measured with fine digital callipers, and tumour volume was calculated by using the following formula: tumour volume = (length × width^2^)/2. After the tumour volume reached approximately 50 mm^3^, the mice were divided into four groups randomly and injected different solutions into tumours of each group: Control group (50 µl double distilled water), IL-35 group (50 µl rIL-35, 0.25 mg/ml, Sino Biological lnc., China), IL-35 NA group (50 µl IL-12A, 0.25 mg/ml, Abcam, USA) and Scramble group (50 µl PBS, ZSJQB Co., Ltd. Beijing., China). Each group had 15 mice, and the survival of the mice was recorded every day. After 2 weeks, all the mice were sacrificed. Isoflurane inhalation was used for anaesthesia. Carbon dioxide asphyxiation followed by cervical dislocation was performed for killing. The tumours were collected, and the tumour volumes were measured. Tumour tissues were dissected and partly formalin fixed and paraffin embedded for immunohistochemistry while the remaining tumours were snap-frozen in liquid nitrogen, then lysed for western blot. The peripheral blood was harvested for ELISA and flow cytometry analysis. Lung tissues were collected and formalin fixed and paraffin embedded for HE staining. Spleens were harvested for flow cytometry analysis.

### Measurement of the IL-35 concentrations

The IL-35 concentrations in plasma were measured by using a commercial mouse IL-35 ELISA kit (Cloud-Clone Corp, Wuhan, People’s Republic of China) according to the manufacturer’s protocol. Each sample was assayed three times. The minimum detectable dose of this kit is typically less than 3.3 pg/mL.

### Detection of lung metastasis

The excised lung tissues were fixed in 10% buffered formalin for 48 h and embedded in paraffin. The lung tissues were sectioned and then stained with HE. Incidence of lung tumour metastasis was examined at day 14.

### Immunohistochemical staining

Mouse tumour tissues were fixed in 10% buffered formalin for 48 h and embedded in paraffin. Sections were deparaffinized and rehydrated. For immunohistochemical staining (IHC), antigen retrieval and endogenous peroxidase inhibition were performed. The sections were then blocked with foetal calf plasma, incubated with mouse anti-p35 antibody (ab66064, Abcam), anti-IL12RB2 antibody (ab203209, Abcam), anti-CD31 antibody (ab28364, Abcam), anti-Ki67 antibody (ab15580, Abcam), anti-CD11b antibody (ab133357, Abcam), anti-Ly6g antibody (ab238132, Abcam), anti-FOXP3 antibody (ab215206, Abcam), anti-CD4 antibody (ab183685, Abcam), and anti-CD8 antibody (ab217344, Abcam), and then with secondary antibody. The sections were visualized with 3,3-diaminobenzidine (DAB) and Mayer’s haematoxylin staining. The intensity score was evaluated blindly by three independent observers. The intensity score was defined as 0, 1, 2, 3, or 4 for negative, weak, moderate, strong or extremely strong staining, respectively. CD31 staining was used to mark the endothelial cells. Large and small microvessels as well as single brown immunostained endothelial cells were included in the microvessel count as recommended in consensus guidelines [19]. The microvessel density (MVD) was calculated by determining the average microvessel counts of 10 random fields at 100× magnification per section. Images were generated using ImageScope Viewer (Leica Biosystems).

### Flow cytometry and intracellular staining

Single-cell suspensions were prepared from the spleens and peripheral blood of mice. Spleens were mechanically disrupted using the plunger end of a 10 ml syringe and resuspended in 1% FBS/PBS. Peripheral blood in centrifuge tubes was placed into a 4℃ high speed centrifuge and spun at 1500 rpm for 5 min. The supernatant was frozen at -20℃ for subsequent ELISA detection. Then, 2 ml of erythrocyte lysate was added to the precipitate of the centrifuge tube, vibrated and incubated at room temperature for 10 min. Then, the tubes were centrifuged, the supernatant was discarded, and the cells were resuspended in 1% FBS/PBS.

Zombie NIR Fixable Viability Kit (Biolegend, USA) were used to stain dead cells. For cell-surface markers, single-cell suspensions were harvested and incubated with anti-mouse/human CD11b-PE, anti-mouse Ly-6G/Ly-6C (Gr-1)-APC, anti-mouse CD4-FITC, anti-mouse CD8a-PE, anti-mouse CD25-APC, anti-mouse FOXP3-PE or appropriate isotype controls (Biolegend, USA) for 30 min at 4 °C. Fixation buffer was added for 20 min, then cells were resuspended in 1% PBS. Finally, all samples were analysed by flow cytometry, and data analysis was processed using FlowJo V10 (Treestar, Inc.).

### Statistical analysis

All statistical analyses were carried out by using SPSS 23.0 software (IBM Corporation, Armonk, NY, USA) and GraphPad Prism 7.00 (GraphPad Software, La Jolla, CA, USA). Data are expressed as the mean ± standard deviation (SD). Continuous variables were measured by the Kolmogorov–Smirnov test to fit the data to a normal distribution. Student’s t-test was used to test parametric data, and Kruskal–Wallis test was used to test non-parametric data. Two-way ANOVA was used to analyse cell growth data. The lung metastasis rate was compared by Fisher’s exact test. Two -sided p-values were calculated, and *p* < 0.05 was considered statistically significant. Kaplan–Meier curves were analysed for relevant variables. The log-rank test was used to analyse the differences in survival times among different groups. Spearman’s correlation analysis was applied to test the correlation of EBI3 and p35 in PCA patients.

## Results

### IL-35 expression in PCA cell lines

**Fig. 1:**
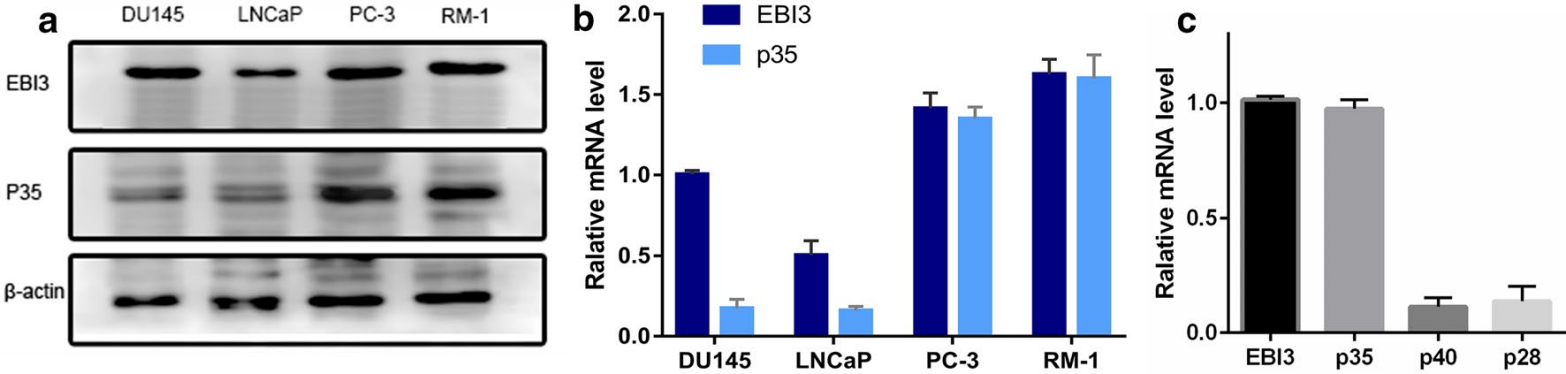
IL-35 expression in DU145, LNCaP, PC-3 and RM-1 cells. **a** EBI3 and p35 expressions in four PCA cell lines. EBI3 and p35 expression levels were detected by Western blot in DU145, LNCaP, PC-3 and RM-1 cells. **b** The mRNA levels of EBI3 and p35 in four PCA cell lines. Quantification of EBI3 and p35 mRNA determined by realtime PCR. The mRNA levels of EBI3 and p35 was high in PC-3 cells and RM-1 cells. **c **Quantification of EBI3, p35, p40 and p28 mRNA in RM-1 cells determined by realtime PCR**.** The mRNA levels of EBI3 and p35 was high, while mRNA levels of p40 and p28 were low in RM-1 cells

First, we investigated the expression of IL-35 in four PCA cell lines, at the same time, to screen the appropriate PCA cell lines for the in vitro study. The two subunits of IL-35 were detectable in all four cell lines, and higher IL-35 levels were observed in PC-3 and RM-1 cells, especially in RM-1 cells. This was verified by western blot (Fig. 1a) and real-time RT-PCR (Fig. 1b). EBI3 was highly expressed in DU145, LNCaP, PC-3 and RM-1 cells, but p35 was only highly expressed in PC-3 and RM-1 cells. Then, we detected the expression of EBI3, p35, p40 (IL-12 is composed of p35 and p40) and p28 (IL-27 is composed of p28 and EBI3). As a result, EBI3 and p35 were high expressed in RM-1 cells, but the expression of p40 and p28 were relatively low (Fig. 1c). Based on the above results, RM-1 cell lines were chosen for subsequent experiments.

### IL-35 enhances malignant biological behaviour of RM-1 cells in vitro

**Fig. 2:**
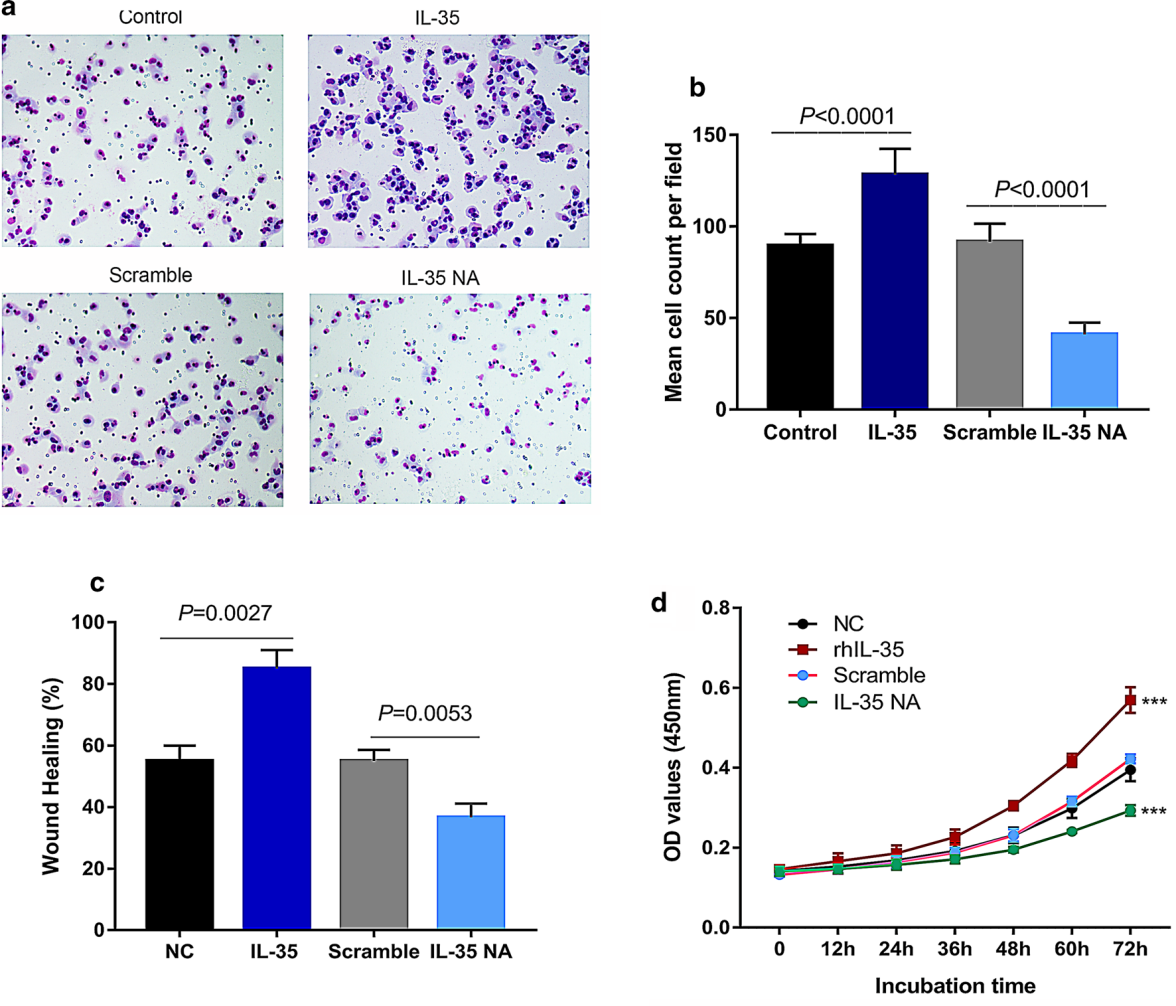
The invasion, migration and proliferation of RM-1 cells. **a** The cells that penetrated the matrix-coated membrane. The ability of invasion was examined by transwell assay. **b** Mean cell count (per field) that penetrated the matrix-coated membrane. The results are reported as the mean of triplicate assays. IL-35 promoted invasion of RM-1 cells and IL-35 neutralizing antibody decreased RM-1 invasion. **c** The migration ability of RM-1 cells was detected using the wound healing assay. Cell migration was calculated as the area of cell migration compared to the area of the initial wound. The results are reported as the mean of triplicate assays. **d** The proliferation of RM-1 cells were examined by CCK-8 assay. The experiment was independently performed three times. ***p < 0.001

To further investigate the biological behaviour of IL-35 in PCA cells, cell migration, invasion, and proliferation abilities were analysed in RM-1 cell lines. The transwell assay indicated that cell invasion ability increased with rIL-35 protein treatment (*p* < 0.001), but decreased with IL-35 neutralizing antibody treatment (*p* < 0.001) (Fig. 2a, 2b). We evaluated the migration behaviour of RM-1 cells exposed to IL-35 using a wound healing assay (Fig. 2c). After exposure for 48 h, the percentage of wound closure in IL-35-exposed cells was higher than that in the control (*p* = 0.0027). The migration distance of RM-1 cells treated with IL-35 neutralizing antibody was closer than that of the scramble group (*p* = 0.0053). The CCK‐8 assay was applied to investigate cell proliferation (Fig. 2d). Compared to the negative control group, cell growth was significantly promoted, as evidenced in cells after exposure to rIL-35 protein (*p* = 0.002). Cell growth was remarkably inhibited after IL-35 neutralizing antibody administration using the scramble group as a control (*p* = 0.0002).

### IL-35 promotes progression of PCA in vivo

**Fig. 3:**
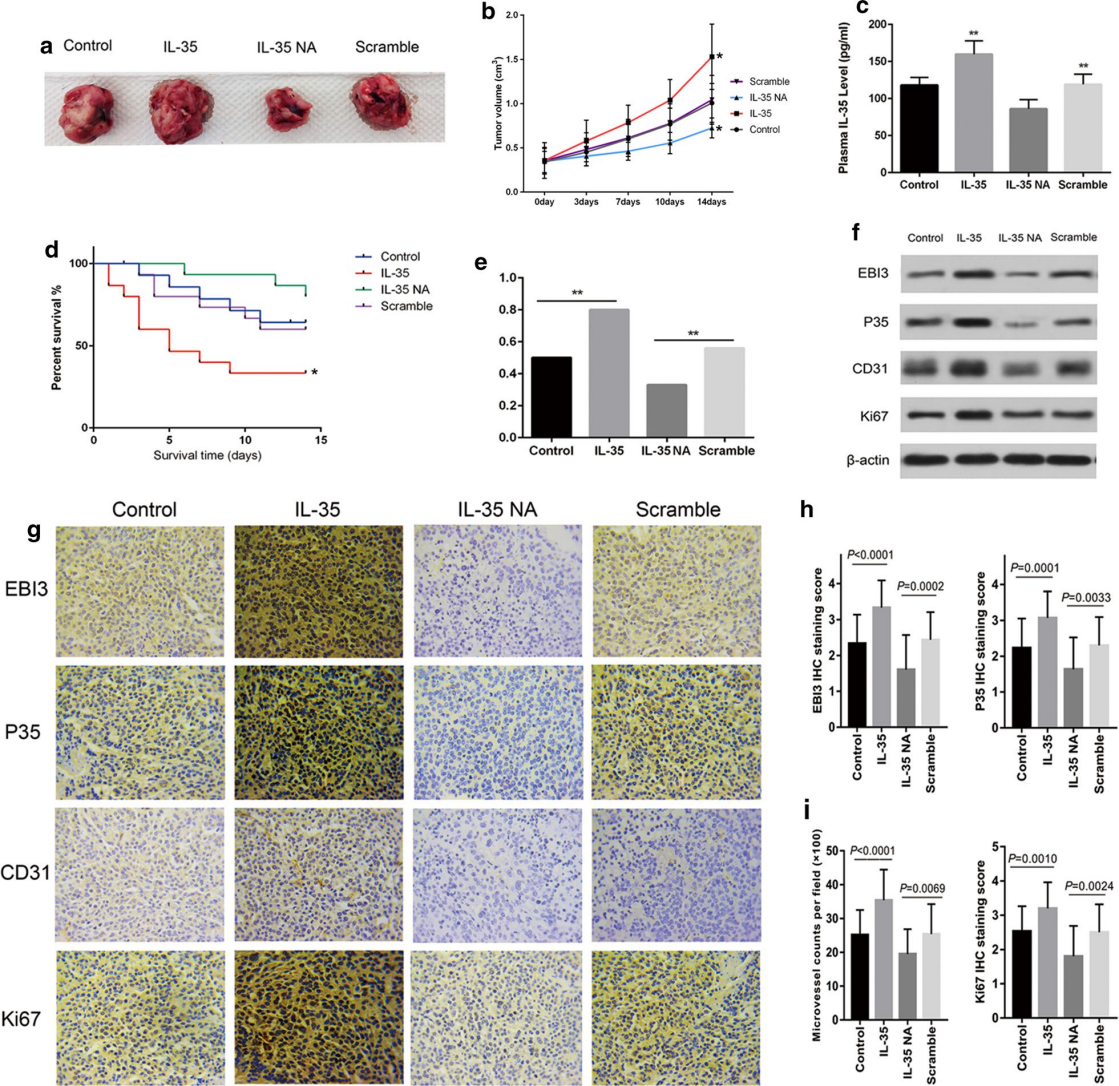
Experimental results in mice. **a** Tumors image in four groups. **b** Growth curves of tumors of indicated groups. *p < 0.05. **c** Concentration of IL-35 in mice plasma of indicated groups. **p < 0.01. **d** Survival curves of mice in four groups. *p < 0.05. **e** Detection of metastatic PCA in the excised lung tissue by HE staining. Incidence of lung tumor metastasis was examined at day 15. *p* value was determined using Fisher’s exact test. **p < 0.01. **f** EBI3, p35, CD31 and Ki67 expressions in tumor tissues. **g** IHC staining for CD31 was performed to determine the MVD in tumor tissues. Ki67 represents the ability of cell proliferation. Representative images of IHC staining for EBI3, IL12A, CD31 and Ki67. (×200). **h** EBI3 and p35 staining scores in each group. **i** Left, the microvessel counts per field in each group (×100). Right, Ki67 staining scores in each group. Data are presented as mean ± SD

To investigate the role of IL-35 in tumour growth in mice, a xenograft PCA model was used. Images and growth curves of tumours in four groups are shown in Fig. 3a, b. The tumour volume of mice in the IL-35 group was significantly larger than that in the NC group (1.53 ± 0.27 vs. 1.01 ± 0.22 cm^3^; *p* = 0.014), whereas the IL-35 NA group was significantly smaller than that in the scramble group (0.73 ± 0.11 vs. 1.04 ± 0.28 cm^3^; *p* = 0.025). ELISA results showed that the mean IL-35 plasma concentration in the IL-35 group was significantly higher than that in the control group (159.87 ± 18.02 vs. 118.37 ± 10.39 pg/ml, respectively; *p* = 0.0006; Fig. 3c), and the mean IL-35 plasma concentration in the IL-35 NA group was markedly lower than that in the scramble group (86.13 ± 12.53 vs. 119.09 ± 13.70 pg/ml; *p* = 0.0014; Fig. 3c). Furthermore, the overall survival rate of the IL-35 group was evidently decreased compared with the control group (*p* < 0.05), but the overall survival rate of the IL-35 NA group was slightly higher than that of the scramble group (*p* = 0.18; Fig. 3d). In addition, the lung metastasis rate in the IL-35 group was 80.00%, which was remarkably higher than that in the control group (50.00%, *p* = 0.001). The lung metastasis rate in the IL-35 NA group was 33.30%, which was remarkably lower than that in the scramble group (55.56%, *p* = 0.002; Fig. 3e).

### IL-35 facilitates angiogenesis and cell proliferation in tumour tissues from mice

Western blot and immunohistochemistry results indicated that EBI3 and p35 were highly expressed in the tumours of the IL-35 group and less expressed in those of the IL-35 NA group (Fig. 3f–h). A CD31 antibody was used to stain vascular endothelial cells and to calculate the micro vessel density (MVD). The MVD in tumour tissues with high IL-35 levels from the IL-35 group was significantly increased compared with the control group (35.40 ± 1.65 vs 25.17 ± 1.34; *p* < 0.0001; Fig. 3i). The MVD in tumour tissues with low IL-35 levels from the IL-35 NA group was significantly decreased compared with the scramble group (19.47 ± 1.35 vs 25.37 ± 1.68; *p* = 0.0069; Fig. 3h). This result was further validated by western blot (Fig. 3f). In addition, Ki67 was highly expressed in the IL-35 group compared with the control group, and less expressed in the IL-35 NA group compared with the scramble group (Fig. 3f, g, i).

### IL-35 effects on MDSCs, Tregs, and CD4+ and CD8+ T cells in the tumour microenvironment

**Fig. 4:**
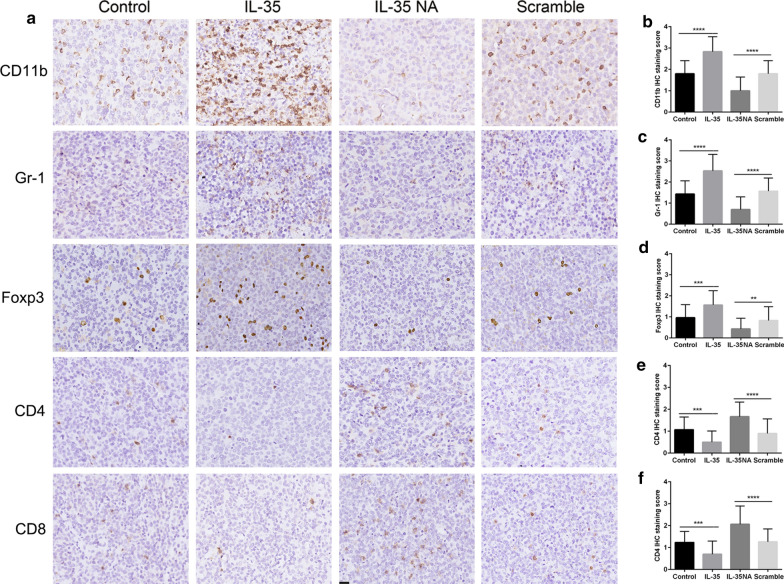
IHC staining for CD11b, Gr-1, Foxp3, CD4 and CD8 in tumour tissue. **a** Representative images of IHC staining for CD11b, Gr-1, Foxp3, CD4 and CD8. **b** CD11b staining scores in each group. **c** Gr-1 staining scores in each group. **d** Foxp3 staining scores in each group. **e** CD4 staining scores in each group. **f** CD8 staining scores in each group. Data are presented as mean ± SD. **p < 0.01, ***p < 0.001, ****p < 0.0001

The immunohistochemistry results indicated that CD11b, Gr-1 and Foxp3 were highly expressed in the tumours of the IL-35 group and less expressed in those of the IL-35 NA group (*p* < 0.01; Fig. 4a, b–d). CD4 and CD8 were less expressed in the IL-35 group compared with the control group, and highly expressed in the IL-35 NA group compared with the scramble group (*p* < 0.001; Fig. 4a, e, f).

### IL-35 effects on MDSCs, Tregs, and CD4+ and CD8+ T cells in spleen and blood

**Fig. 5:**
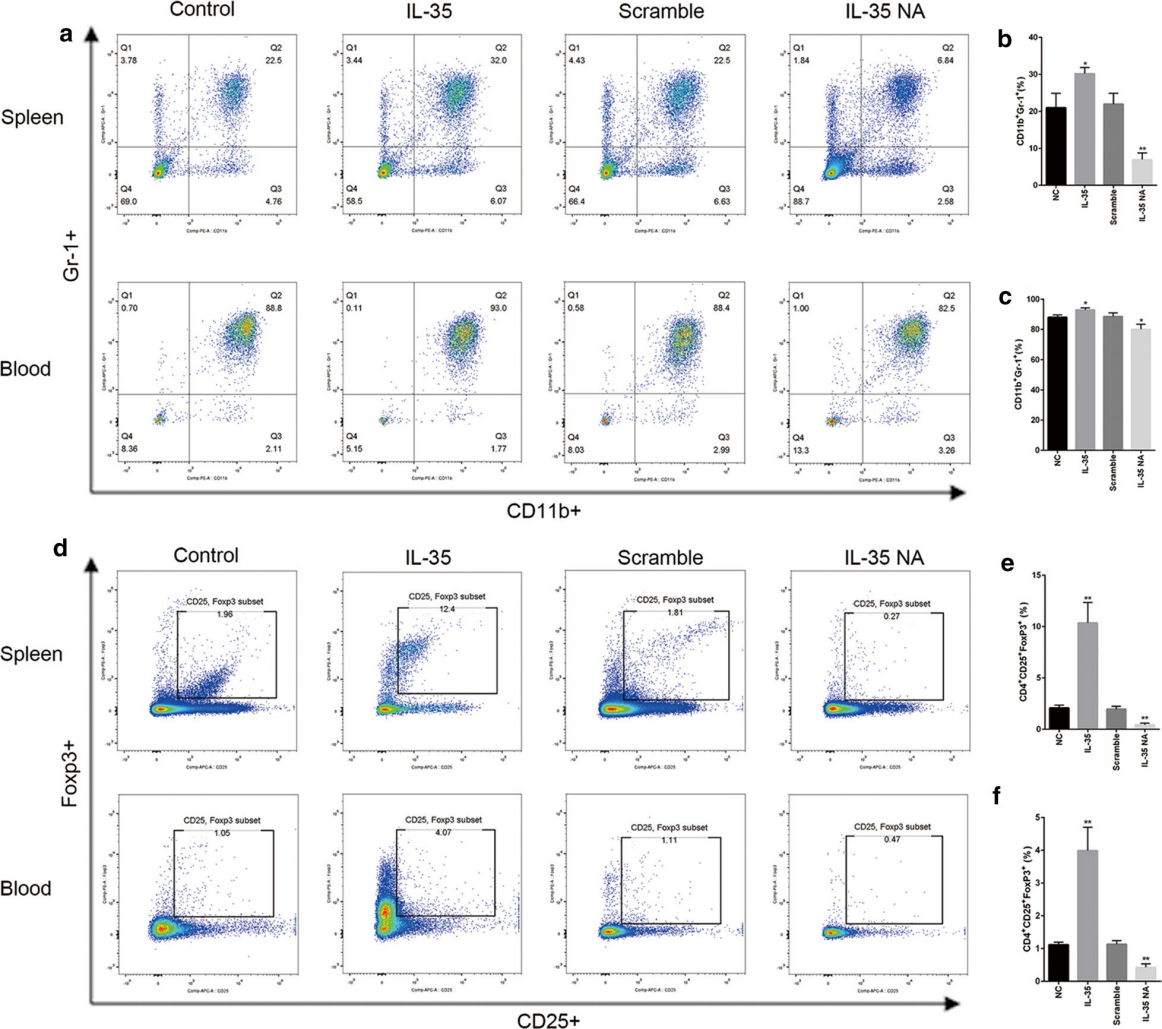
Changes of MDSC and Treg cell number in the spleen and blood. **a** The MDSCs (CD11b+ Gr-1+) percentages in the whole cell counts of spleen and blood were detected by flow cytometry. **b** The percentages of MDSCs (CD11b+ Gr-1+) in spleen were statistically analyzed. **c** The percentages of MDSCs (CD11b+ Gr-1+) in blood were statistically analyzed. **d** The Treg cells (CD4+ CD25+ Foxp3+) percentages in the whole cell counts of spleen and blood were detected by flow cytometry. **e** The percentages of Treg cells (CD4+ CD25+ Foxp3+) in spleen were statistically analyzed. **f** The percentages of Treg cells (CD4+ CD25+ Foxp3+) in blood were statistically analyzed. Data are presented as mean ± SD and significance levels are indicated as follows: *p < 0.05, **p < 0.01

As shown in Fig. 5, we found that high levels of IL-35 significantly increased the proportion of MDSCs in spleen (30.20% ± 0.96% vs 20.97% ± 2.27%; *p* = 0.0200) and blood (92.87% ± 0.75% vs 87.97% ± 0.94%; *p* = 0.0151), whereas low levels of IL-35 significantly decreased the proportion of MDSCs in spleen (6.93% ± 1.05% vs 21.97% ± 1.70%; *p* = 0.0017) and blood (80.03% ± 1.94% vs 88.50% ± 1.36%; *p* = 0.0233). In addition, the percentage of Treg cells in the IL-35 group was increased in both spleen (10.36% ± 1.15% vs 20.97% ± 2.07%; P = 0.0020) and plasma (3.99% ± 0.41% vs 1.11% ± 0.04%; *p* = 0.0023). In the IL-35 NA group, the percentage of Treg cells was evidently decreased in both the spleen (0.44% ± 0.09% vs 1.96% ± 0.16%; *p* = 0.0011) and plasma (0.42% ± 0.06% vs 1.13% ± 0.06%; *p* = 0.0012).

**Fig. 6:**
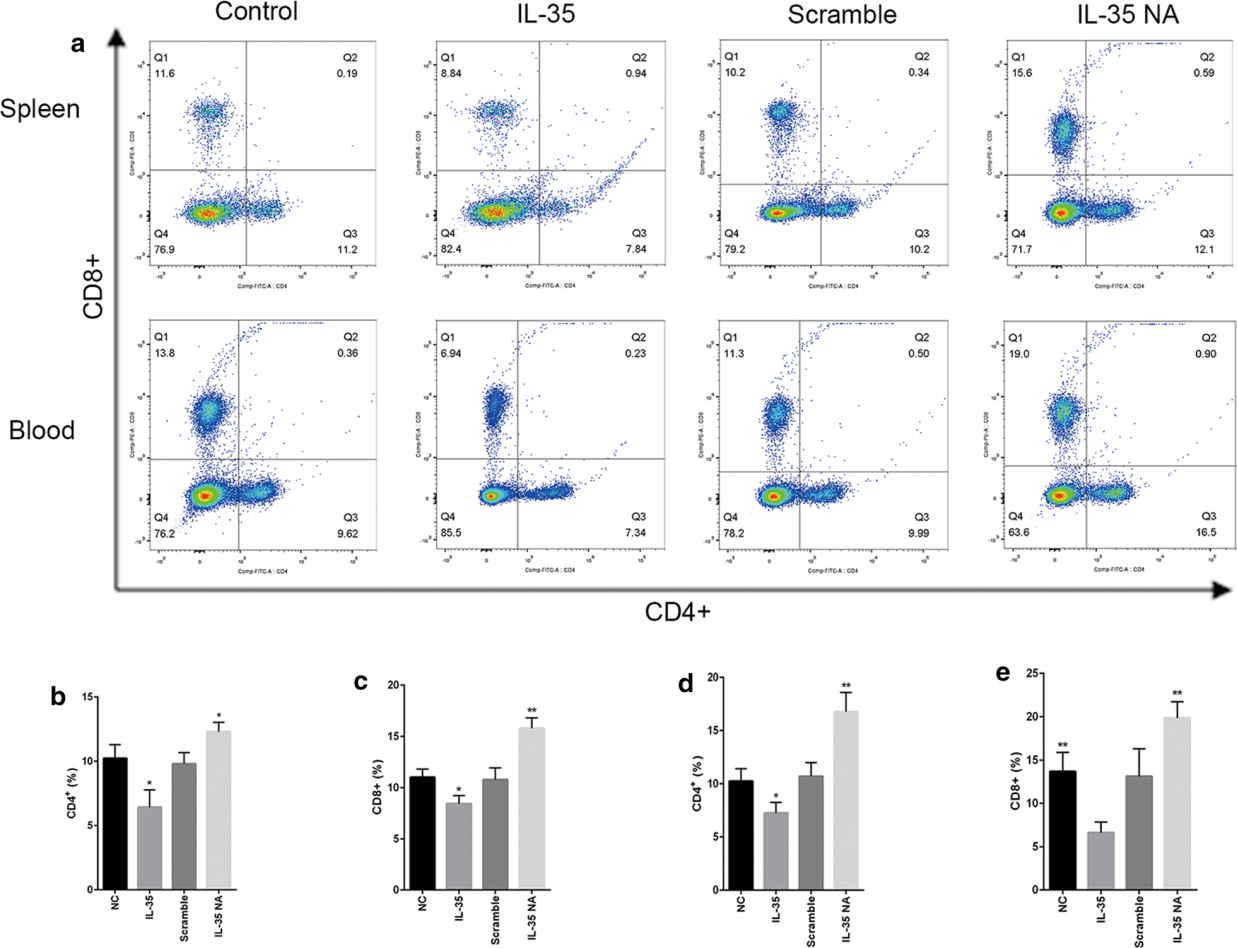
Changes of CD4+ T cell and CD+ T cell number in the spleen and blood. **a** The CD4+ and CD8+ T cells percentages in the whole cell counts of spleen and blood were detected by flow cytometry. **b**, **c** The percentages of CD4+ and CD8+ T cells in spleen were statistically analyzed. **d**, **e** The percentages of CD4+ and CD8+ T cells in blood were statistically analyzed. Data are presented as mean ± SD and significance levels are indicated as follows: *p < 0.05, **p < 0.01

Furthermore, the IL-35 group showed a decrease in CD4+ and CD8+ T cells compared with the control group, and the IL-35 NA group showed an increase in CD4+ and CD8+ T cells compared with the scramble group, both in the spleen and in the blood (Fig. 6).

## Discussion

IL-35 is a member of the IL-12 cytokine family, which has been detected in a variety of diseases and different cell types [20–23]. IL-35 has led many scholars to study its expression regulation, molecular signalling and immunoregulation. Recent studies have shown that IL-35 is overexpressed in a variety of tumours [24–27]. It can be used directly in tumour cells to regulate the expression of related proteins, and it also plays an important role in the occurrence and development of tumours by inducing the production of regulatory T cells, inhibiting the response of effector T cells, regulating anti-tumour immune response and inducing immune escape [10, 28, 29]. Therefore, IL-35 has become a promising biomarker and a target for tumour therapy.

Our previous work showed that plasma IL-35 expression levels in PCA patients were significantly increased compared with those in both patients with benign prostatic hyperplasia and healthy controls [17]. The level of IL-35 was associated with the progression and metastasis of PCA patients [17, 30]. However, the effect of IL-35 on the progression of PCA and its related mechanism are not clear. To the best of our knowledge, this is the first study to detect the effects of IL-35 on the proliferation, metastasis, angiogenesis and prognosis of PCA in vitro and in vivo. In addition, this is also the first exploration into the impact of IL-35 on the immune cells of PCA mice and analysis of the possible mechanisms involved.

In the current study, we first investigated the expression of IL-35 in four kinds of PCA cell lines that were widely used in prostate cancer research, including human PCA cell lines LNCaP, DU145, PC-3, and mouse PCA cell line RM-1. LNCaP was established by Horoszewicz et al. from metastatic lymph nodes of PCA patients in 1980 [31]. It retains the characteristics of the early differentiation function of PCA cells and has the obvious characteristics of androgen-dependent prostate cancer. DU145 was isolated from brain metastases of PCA with a relatively low degree of differentiation and great potential for metastasis [32]. Its androgen dependence needs to be further confirmed [33]. PC-3 was isolated from human PCA bone metastases with low differentiation and no endogenous androgen receptor. It is a non-androgen-dependent prostate cancer cell with high intensity metastasis potential [32]. In this study, EBI3 and p35 were all highly expressed in PC-3 cells, but only EBI3 was highly expressed in the hormone-sensitive LNCaP cell line and the moderately malignant DU145 cell line, indicating that IL-35 might be related to the malignancy and progression of PCA.

Next, we treated mouse PCA RM-1 cells with DD water, rIL-35 protein, PBS and IL-35 neutralizing antibody, respectively. We found that IL-35 promoted migration and invasion of RM-1 cells by transwell and wound healing assays. IL-35 neutralizing antibody (anti-IL-12A antibody) suppressed migration and invasion of PCA cancer in vitro. This conclusion was further verified in animal experiments. The lung metastasis rate in the IL-35 group with high plasma IL-35 concentration was significantly higher than that in the control group, while the lung metastasis rate in the IL-35 NA group with low IL-35 concentration was significantly lower than that in the scramble group. Based on the above results in the present study, IL-35 played a role in promoting metastasis of PCA, and it might be a target for reducing metastasis of PCA. Similarly, Huang et al*.* suggested that IL-35 played an important role in the invasion and metastasis of pancreatic ductal cancer (PDAC) cells [34]. Its mechanism was that IL-35 promoted the overexpression of ICAM1 through the gp130-STAT 1 signalling pathway to improve endothelial adhesion and transendothelial migration of PDAC cells [34].

To explore the influence of IL-35 on the proliferation of PCA, we carried out a CCK-8 assay in vitro. Our results showed that IL-35 promoted the proliferation of RM-1 cells, and the IL-35 neutralizing antibody played the opposite role. This was further validated in an animal study. High levels of IL-35 promoted the growth of PCA tumours in mice, while reduced IL-35 levels restrained tumour growth in vivo. Additionally, Ki67, which is a marker of cell proliferation, was highly expressed in the tumours of the IL-35 group and was expressed at low levels in the IL-35 NA group. These results suggested that IL-35 facilitated cell proliferation of PCA and that decreasing IL-35 was effective at inhibiting the cell proliferation of PCA in vivo. This result is consistent with other studies about IL-35 in breast, colon and pancreas cancers [35–37].

The results of experiments in vivo showed that the overall survival rate of mice overexpressing IL-35 in blood and tissue was significantly lower than that of the control group, which indicated that Il-35 was of great clinical significance in evaluating the prognosis of PCA. The overexpression of IL-35 in tumour tissue and plasma is closely related to tumour progression and poor prognosis in several kinds of cancers. The high expression of IL-35 in pancreatic ductal adenocarcinoma was positively correlated with TNM stage and vascular invasion [38]. IL-35 was highly expressed in the plasma of patients with non-small cell lung cancer (NSCLC) and negatively correlated with survival time [15]. IL-35 was expressed and secreted in breast cancer cells, which was related to poor prognosis of patients and was an independent prognostic factor [35]. The concentration of serum IL-35 and the presence of IL-35 in tumours were positively correlated with the clinical stage of colorectal tumours [26, 39]. Surgical resection of tumours resulted in a decrease in serum IL-35 concentrations, indicating that this cytokine originated from tumours and could be used as an important biomarker for evaluating tumour progression [13, 39, 40].

It is generally accepted that tumour angiogenesis is crucial for tumour growth. The CD31 protein is present on endothelial cells in microvessels. High CD31 expression is closely related to advanced disease and poor survival in many kinds of cancers [41, 42]. Our results showed that IL-35 significantly increased the expression of CD31 in prostate cancer tissues of mice compared with the control group. It was suggested that IL-35 might promote the malignant development of PCA by upregulating CD31 expression and promoting tumour angiogenesis. Meanwhile, tumours in mice with low IL-35 expression had fewer CD31 expression. This result showed that anti-IL-35 treatment played an important role in inhibiting tumour angiogenesis. The results of this study are similar to those of Huang Chongbiao et al. in pancreatic cancer [37], but the mechanism is different. They found that IL-35 increased the aggregation of monocytes and the expression of CXCL-1 and CXCL-8, which promoted angiogenesis in pancreatic ductal carcinoma. Combined with gemcitabine, the anti-IL-35 neutralizing antibody could significantly reduce monocyte infiltration, microvessel density and volume of PDAC tumour [37].

Treg cells have a vital effect on inhibiting antitumour immunity by hindering the protective immune surveillance of the tumour, inducing immune escape, and blocking the effective antitumour immune response of the tumour-bearing host [4]. In the context of tumours, a large number of Treg cells were often detected, which inhibited or reduced these effector lymphocytes, such as CD4+ T cells and CD8+ cytotoxicity T lymphocytes (CTL), achieving antitumour immune responses [43–45]. CD4+ T cells were reported to be helper T cells, which are capable of promoting effective antitumour immune responses. CD8+ T cells mainly refer to CTL, and the increase of CD8+ CTL could kill the tumours efficiently. The results of this study showed that IL-35 could significantly increase the proportion of CD4+ Foxp3+ Treg cells and decrease the proportion of CD4+ and CD8+ T in the spleen, blood and tumour tissue of mice with prostate cancer, indicating that IL-35 could promote CD4+ Foxp3+ Treg cells, inhibiting antitumour immunity and promoting the progression of PCA. The proportion of Treg cells in the IL-35 neutralizing antibody group was decreased, and the proportions of CD4+ and CD8+ T cells were increased significantly, indicating that the IL-35 neutralizing antibody might restrain the progression of PCA by decreasing Treg cells.

MDSCs, known as superantigen-derived cells, played an important role in promoting tumour survival, growth and drug resistance [46]. MDSCs are a group of heterogeneous cells composed of activated immature cells and their precursors. Stimulated by tumour cells and matrix-released factors, leukocyte precursors cannot differentiate into normal immune cells. MDSCs, as immature cells, could leave their site of origin, migrate throughout the whole body, and mediate immunosuppression [46]. Previous studies have shown that tumour-derived IL-35 could increase the accumulation of CD11+ Gr1+ MDSCs in the tumour microenvironment, thus promoting tumour angiogenesis [9]. The tumour cells treated with IL-35 showed decreased sensitivity to the destruction of CTLs, thus inhibiting anti-tumour immunity [9]. The results of our study showed that IL-35 could significantly increase the proportion of MDSCs in the spleen, blood and tumour tissue of mice with PCA, indicating that IL-35 could promote the proliferation of MDSCs, thus inhibiting anti-tumour immunity and promoting the progression of PCA. The proportion of MDSCs in the IL-35 neutralizing antibody group decreased significantly, indicating that IL-35 might be a new target for the treatment of PCA.

However, there are some limitations in present study. Since no conformational antibodies specific to IL-35 are available at present, we detected the expression of EBI3 and p35 for the IHC staining standing for IL-35. We will do further verification if better IL-35 antibodies are available in the future. Another question is that the ways IL-35 promotes cell proliferation and tumour angiogenesis in PCA are still unclear. We will explore more specific mechanisms in further investigations.

## Conclusion

In conclusion, IL-35 contributed to the progression and metastasis of PCA by promoting cell proliferation and tumour angiogenesis. IL-35 might limit the anti-tumour immune response by upregulating the proportions of Tregs and MDSCs and by reducing the proportions of CD4+ and CD8+ T cells. These findings provide new insight into the function of IL-35 in the progression of PCA and underscore the potential significance of IL-35 as a therapeutic target for PCA.

## Data Availability

The datasets used and/or analyzed during the current study are available from the corresponding author on reasonable request.

## Abbreviations

CCK-8: Cell-counting kit-8
CTL: Cytotoxicity T lymphocyte
CXCL: CXC chemokine ligands
EBI3: Epstein Barr virus induced 3
Foxp3: Forkhead box p3
IL: Interleukin
MDSCs: Myeloid-derived inhibitory cells
PCA: Prostate cancer
RPMI: Roswell park memorial institute
RT-PCR: Real-time polymerase chain reaction
SPF: Specific pathogen-free
TGF: Transforming growth factor
Tregs: Regulatory T cells
